# Design of thermoelectric materials with high electrical conductivity, high Seebeck coefficient, and low thermal conductivity

**DOI:** 10.1002/ansa.202000114

**Published:** 2020-12-03

**Authors:** Hiroki Yoshihama, Hiromasa Kaneko

**Affiliations:** ^1^ Department of Applied Chemistry School of Science and Technology Meiji University Kawasaki Kanagawa Japan

**Keywords:** machine learning, material design, QSPR, thermoelectric materials, XRD spectra

## Abstract

Thermoelectric materials with a high Seebeck coefficient, high electrical conductivity, and low thermal conductivity are required to directly and efficiently convert unused heat into electricity. In this study, we construct models predicting the Seebeck coefficient, electrical conductivity, and thermal conductivity using existing material databases. In addition to the ratios of atoms in the crystals and temperature at which the materials are used, the values from the X‐ray diffraction (XRD) spectra were used as inputs to represent the crystal structure of the materials. It was confirmed that the constructed models could predict the properties with high accuracy using the X‐ray diffraction values. Additionally, using the constructed models, we succeeded in proposing promising new candidate materials with high Seebeck coefficients, high electric conductivities, and low thermal conductivities.

## 1 INTRODUCTION

Renewable power generation^1^ is one avenue for addressing the energy problems caused by the depletion of petroleum resources and the increasing energy demands associated with a growing global population. Thermoelectric conversion that directly converts unused heat, such as geothermal and industrial waste heat, into electricity is promising because it is not significantly affected by the time of day and variable weather, it does not emit carbon dioxide, and there is little concern about breakdown or maintenance.^2^ The performance of thermoelectric materials^3^ is determined by the dimensionless index *ZT* given as follows:

(1) $$ZT=\frac{S^{2}\sigma }{\kappa }T.$$

Here, *S* is the Seebeck coefficient [μV/K], *σ* is the electrical conductivity [/(Ω˙m)], *κ* is the thermal conductivity [W/(m˙K)], and *T* is the temperature [K]. Materials with a better performance have a larger *ZT* with a higher Seebeck coefficient, higher electrical conductivity, and lower thermal conductivity. In past research, thermoelectric materials were developed by empirically predicting the chemical structures of materials with the three desired properties and evaluating the experimental structures.^4^, ^5^ The issue with this approach is that it relies on the experience and intuition of chemists and the cost and time required can be enormous.

One possible solution is to design the optimal materials using machine learning with an experimental dataset of materials obtained from previous studies. This material design process uses the quantitative structure‐property relationship (QSPR) between the properties and chemical structures of materials to estimate the properties of new chemical structures. It is possible to predict the properties of materials from their chemical structures at a low cost and design materials without relying on the subjective opinions of chemists.

The objective of this study was to construct highly predictive regression models to predict the *σ*, *κ*, *S*, and *ZT* of thermoelectric materials and to search for optimal thermoelectric materials using the models. The objective variables Y are *σ*, *κ*, *S*, and *ZT*, and the explanatory variables X are the ratios of the atoms in the crystals, values of the X‐ray diffraction (XRD) spectra,^6^ and the temperature; then, regression models *Y* = f(*X*) are constructed. The use of the XRD spectra allows us to express the correlation between the crystal structures of materials and their properties, and the use of temperature allows us to propose materials for each temperature. In this study, we developed a direct method of predicting *ZT* and an indirect method by predicting *σ*, *κ*, and *S* and then calculating *ZT* using Equation (1). This was directed at finding new materials for thermoelectric conversion, and the method was adopted with high predictive accuracy.

The predictive ability of the proposed models was validated using a property dataset from the starrydata2 database^7^ and X‐ray diffraction data from the AtomWork‐Adv database.^8^, ^9^ Finally, we proposed promising candidates for use as thermoelectric materials by predicting the previously unknown properties of these materials.

## 2 METHODS

The X‐variables used in constructing regression models are temperature, the ratio of the atoms in the crystals, and the XRD spectra. For temperature, we used the values with the unit K as an X‐variable. The ratios of the atoms in the crystals were also calculated as X‐variables. For example, for the material Cu₂O, the ratio of Cu is 0.66 and O is 0.33. There are 118 variables for the ratios from the element 1 (H) to the element 118 (Og).

**FIGURE 1:**
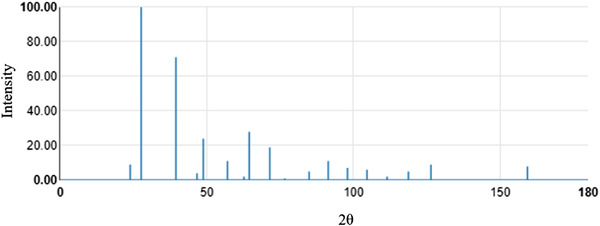
Example XRD spectra in the AtomWork‐Adv database^8^

**FIGURE 2:**
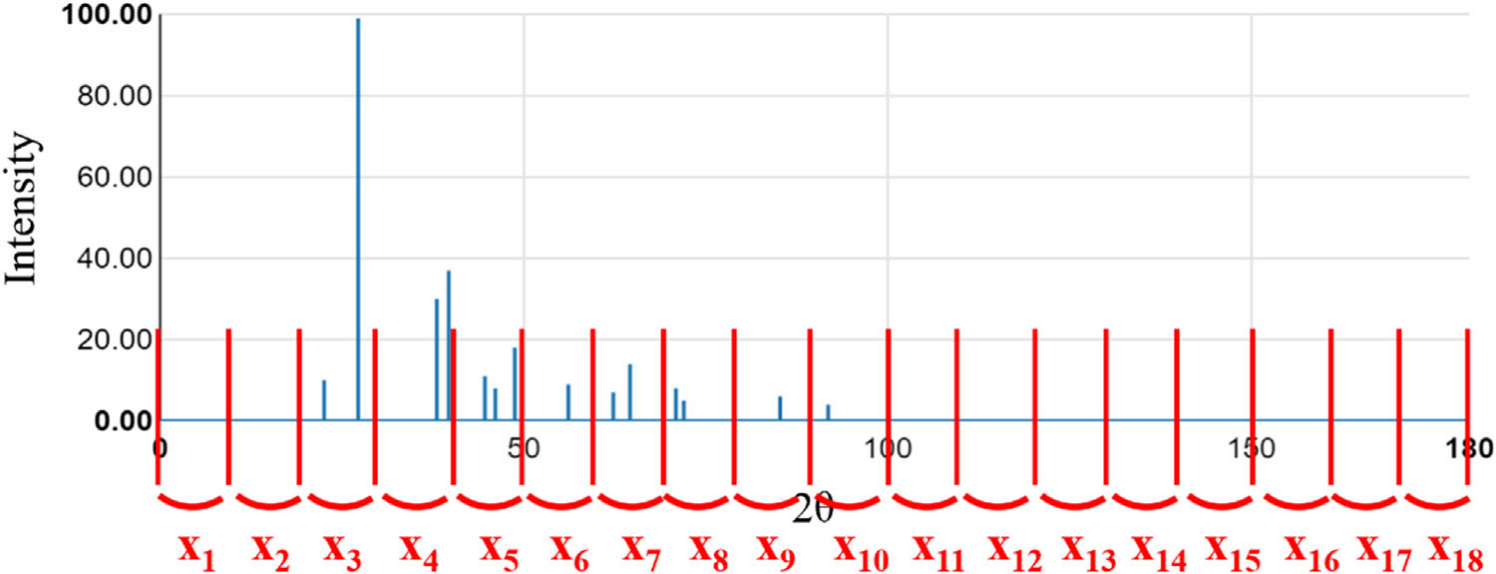
Dividing the XRD spectra into 18 X‐variables (10° unit)

The XRD spectra were calculated using DFT in the AtomWork‐Adv database^8^, ^9^ and were not continuous values. Figure 1 shows an example of XRD spectra in the database. To prepare X‐variables from the spectra, we discuss two methods. The first method is a dividing method (DM; see Figure 2). The diffraction angle (2θ) is divided by a certain delimitation width and the intensity of the diffraction lines within each interval are used as the X‐variables. Ten different widths of 1°, 2°, 3°, 4°, 5°, 6°, 7°, 8°, 9°, and 10° were considered. When more than one diffraction line was present in an interval, three statistics: mean, median, and maximum of the intensities were considered. When no diffractions were present within the interval, the X value of the interval was set as 0. Because there are 10 different widths and 3 statistics, the number of their combinations is 30 (10 × 3).

**FIGURE 3:**
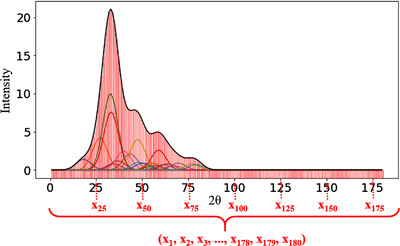
Setting 180 X‐variables using Gaussian functions for the XRD spectra when the standard deviation of the Gaussian functions is 5

The second method was the Gaussian method (GM; see Figure 3). Gaussian functions^10^ were used to represent the spectra. First, the mean of the Gaussian function was set to the value of 2θ for each diffraction line, and the standard deviation was set to a certain value; then, a Gaussian function was generated for each diffraction line. After the Gaussians were added and pseudo XRD spectrum could be obtained like the black line in Figure 3. Then, the X‐variables were values at 180 points in 2θ of the spectrum. For the Gaussian function, 15 different standard deviations of 1, 2, 3, 4, 5, 6, 7, 8, 9, 10, 11, 12, 13, 14, and 15 were considered, and 180 X‐variables were calculated for each standard deviation.

## 3 RESULTS AND DISCUSSION

The validation of the proposed methods was performed using property data from the starrydata2 database^7^ and calculated X‐ray diffraction data from the AtomWork‐Adv database.^8^, ^9^ The total number of samples was 1116; 783 samples were randomly selected as training data and the other 333 samples were test data. Partial least squares regression (PLS)^11^ was used as a linear regression method, and support vector regression (SVR)^12^ and Gaussian process regression (GPR)^13^ were used as nonlinear regression methods to construct the regression models. In SVR, we used the Gaussian kernel given as follows:

(2) $$K\left(x^{\left(i\right)},\;x^{\left(j\right)}\right)=\exp\left(-\gamma {\left\|x^{\left(i\right)}-x^{\left(j\right)}\right\|}^{2}\right),$$

where **x** ^(^ *^i^* ^)^ is X values of the *i*th sample and *γ* is a hyperparameter. In GPR, we used the kernel function given as follows:

(3) $$K\left(x^{\left(i\right)},\;x^{\left(j\right)}\right)=\theta_{0}\exp\left\{-\frac{\theta_{1}}{2}{\left\|x^{\left(i\right)}-x^{\left(j\right)}\right\|}^{2}\right\}+\theta_{2}+\theta_{3}\sum_{k=1}^{m}{x_{k}}^{\left(i\right)}{x_{k}}^{\left(j\right)},$$

where *θ*_0_, *θ*_1_, *θ*_2_, and *θ*_3_ are hyperparameters.

An applicability domain (AD),^14^, ^15^ which is the data domain in which a model has a predictive ability that is as good as that of the training data, was set with the k‐nearest neighbor algorithm (k‐NN) with k = 5. The 5‐NN distance threshold was set so that 90% of the training data was within the AD.

We compared X‐variables of temperature and ratios of atoms, X‐variables of temperature and XRD spectra, and X‐variables of temperature, ratios of atoms and XRD spectra. Tables 1‐3 show prediction results for *κ*, *σ*, and *S* for the test data inside AD, respectively. Mean absolute error (MAE) is the average of the absolute values of the Y errors, where lower MAE values indicate higher predictive accuracy of the model. We selected width and statistics for DM, and standard deviation for GM, by minimizing MAE. From Tables 1, 2, 3, the MAE had a minimum value in GPR for all Y and all X‐variables, and the addition of XRD spectra reduced the MAE and improved the predictive ability of the models. The predictive performance of DM was higher than that of GM, and the optimal delimitation width and the optimal statistics differed by Y.

**TABLE 1:** Prediction results of κ for test data inside AD

X‐variables		Temperature, compositions of atoms	Temperature, XRD spectra	Temperature, compositions of atoms, XRD spectra
Regression method		GPR	GPR	GPR
DM	Width	–	9°	4°
	Statistics	–	Median	Max
GM	Standard deviation	–	–	–
# of samples inside AD		144	166	162
MAE inside AD		0.312	0.275	0.245

**TABLE 2:** Prediction results of σ for test data inside AD

X‐variables		Temperature, compositions of atoms	Temperature, XRD spectra	Temperature, compositions of atoms, XRD spectra
Regression method		GPR	GPR	GPR
DM	Width	–	2°	2°
	Statistics	–	Median	Median
GM	Standard deviation	–	–	–
# of samples inside AD		144	163	148
MAE inside AD		1.52 × 10^4^	1.39 × 10^4^	1.28 × 10^4^

**TABLE 3:** Prediction results of S for test data inside AD

X‐variables		Temperature, compositions of atoms	Temperature, XRD spectra	Temperature, compositions of atoms, XRD spectra
Regression method		GPR	GPR	GPR
DM	Width	–	5°	5°
	Statistics	–	Median	Mean
GM	Standard deviation	–	–	–
# of samples inside AD		144	163	139
MAE inside AD		14.5	12.2	10.7

**FIGURE 4:**
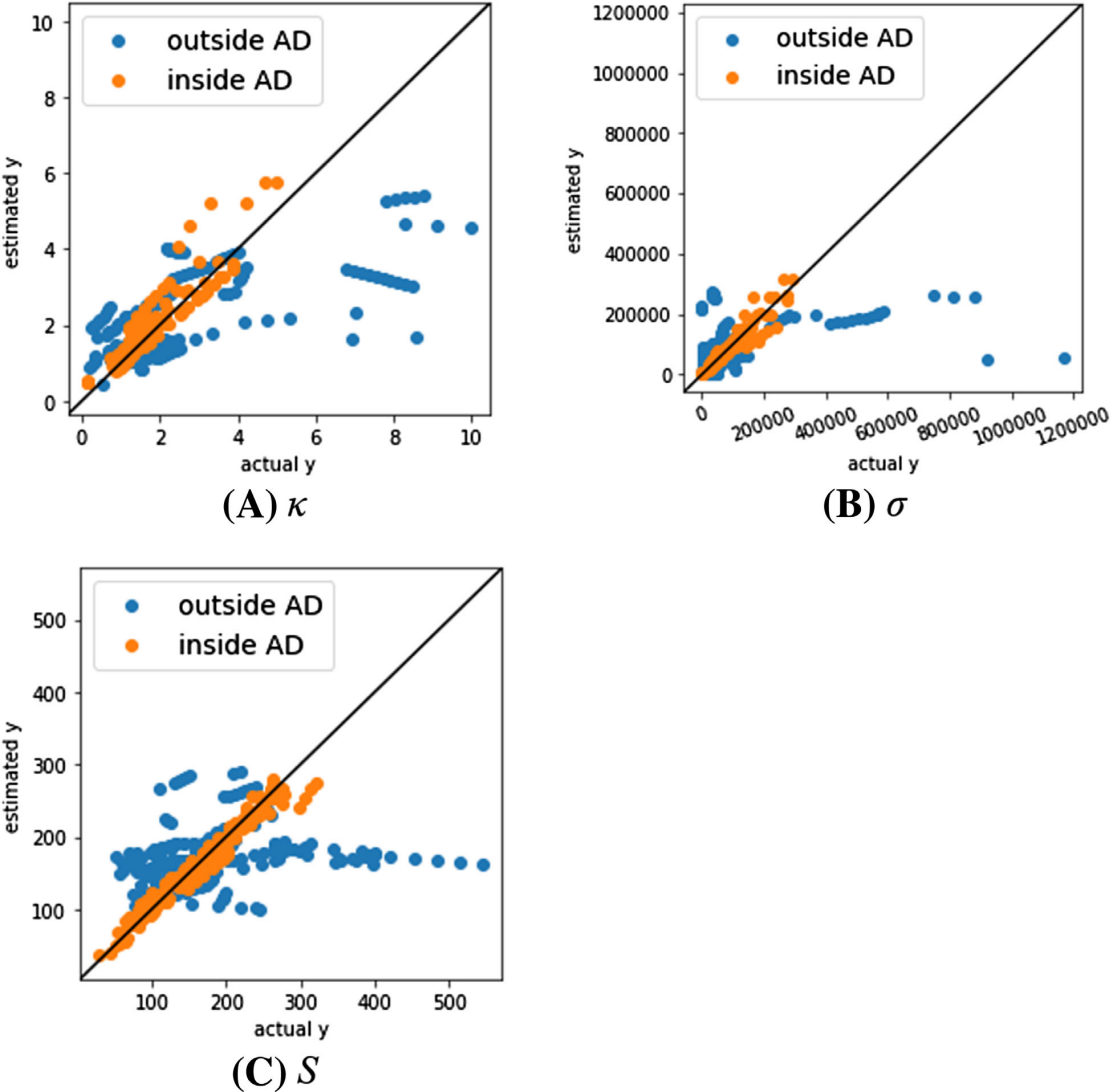
Actual Y vs. estimated Y in κ, σ, and S for test data

Figure 4 shows plots of the measured Y and the estimated Y using test data for the models with the minimum MAE inside AD for each Y. Some of the samples outside the AD were far from the diagonal, while the samples inside the AD were distributed tightly around the diagonal. The proposed method could properly predict Y values for the samples within the AD, and the AD also worked properly.

For the test data within the AD, we compared the predictive performance of the model where *ZT* was predicted directly with that of the model where *ZT* was predicted indirectly from *σ*, *κ* and *S* and Equation (1). When predicting *ZT* directly, the result of the GPR model using only temperature and the ratios of atoms had the lowest MAE in the test data in the AD, with a value of 0.0859. For the case of indirectly predicting *ZT*, the estimated values of *κ*, *σ* and *S* in Figure 4 were converted to *ZT* using Equation (1), and the MAE was 0.0846, which was lower than that of the direct prediction. It was confirmed that the indirect prediction of *ZT* had lower prediction error.

**FIGURE 5:**
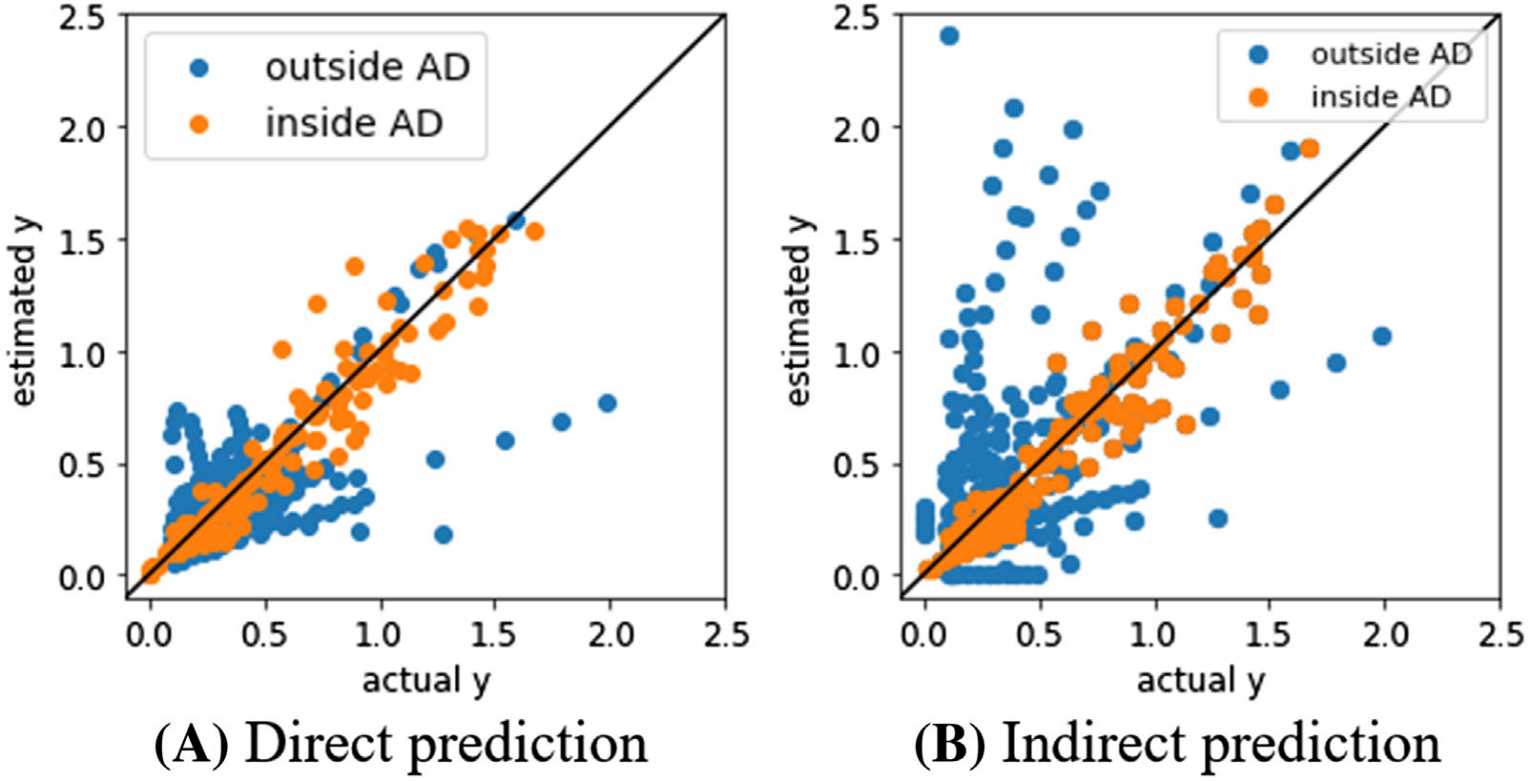
Actual ZT vs. estimated ZT for test data

Figure 5 shows plots of the measured *ZT* and the estimated *ZT* in the test data. Although the average estimation error for the samples inside the AD was lower when *ZT* was predicted indirectly from Table 4, for the samples outside the AD, the samples were closer to the diagonal line when *ZT* was estimated directly. There is a possibility that the extrapolated samples can be predicted more accurately by estimating *ZT* directly.

**TABLE 4:** Prediction results of ZT for test data inside AD

	*r*²	MAE
Direct estimation	0.906	0.0859
Indirect estimation	0.912	0.0846

**FIGURE 6:**
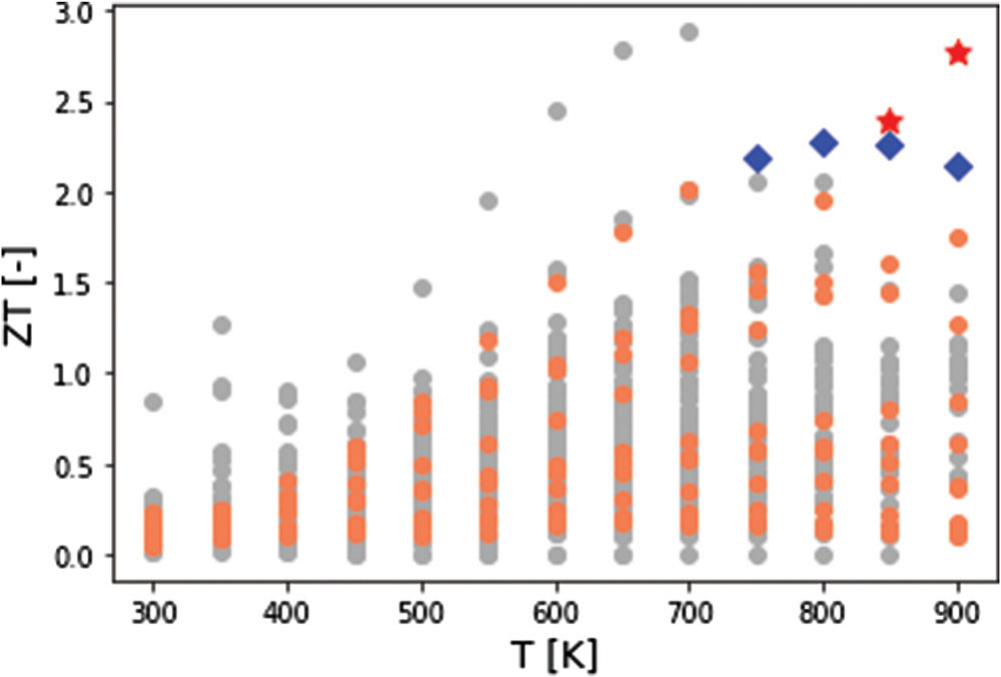
ZT for each temperature. Gray points indicate materials with properties in the database, and the orange points, blue squares, and red stars are new materials. Blue squares are Na0.02Pb0.98Te0.85Se0.1S0.05 and red stars are Na0.02Pb0.98Te0.9Se0.1

Then, using the same method as in Figure 4, we constructed the model for each property with GPR using all samples. Then, 610 materials with unknown properties, i.e., materials with no property base in starrydata2 and AtomWork‐Adv, were prepared. The temperatures were changed to 300 K, 350 K, …, 850 K, and 900 K, and *κ*, *σ*, and *S* were predicted by inputting them into the GPR models. Using the predicted values of *κ*, *σ* and *S*, we predicted *ZT* from Equation (1). The predicted results for *ZT* at each temperature are shown in Figure 6. The gray points indicate materials with properties in the database, and the orange points, blue squares, and red stars indicate new materials. The materials indicated by blue points and red points exceed the *ZT* values of the existing materials at each temperature. The blue squares are Na_0.02_Pb_0.98_Te_0.85_Se_0.1_S_0.05_ and the red stars are Na_0.02_Pb_0.98_Te_0.9_Se_0.1_. The materials with Pb, Te, and Se in the crystal displayed a higher *ZT*. A higher *ZT* was observed for materials with more atoms in the crystal. It was confirmed that the proposed method could be used to design thermoelectric materials that were superior to those in the existing database.

## 4 CONCLUDING REMARKS

This study aimed to design thermoelectric materials with high electrical conductivity, high Seebeck coefficient, and low thermal conductivity using regression models. The models were developed using XRD spectra in addition to the ratios of atoms and temperature as X‐variables for the target properties *ZT*, *σ*, *S*, and *κ*. The reliability of the models could be examined by setting the AD. Analyses of an actual material dataset showed that the use of the XRD spectra improved the predictive ability of the regression models for *σ*, *S*, and *κ*. This indicated that the crystal structure was important for predicting the material's properties and that the XRD spectra could be used to consider the structure. PLS, SVR, and GPR were used as the regression analysis methods, and GPR demonstrated the best predictive performance.

We searched for new thermoelectric materials using the constructed models, and succeeded in proposing new material candidates that are superior to existing materials in the temperature range of 750‐900 K. The results show that it is possible to design promising thermoelectric materials with the appropriate ZT at each temperature on the basis of the ratios of the atoms and the calculated XRD spectra of virtual crystal structures prior to material synthesis. We hope that the proposed method will lead to the practical application of promising thermoelectric materials, which could help address the need for sustainable energy generation.
